# Effect of resin cement type and autoclaving on the retention of zirconia on Ti‐base abutments

**DOI:** 10.1111/jopr.70029

**Published:** 2025-09-20

**Authors:** Carlos Eduardo Sabrosa, Karen Geber

**Affiliations:** ^1^ University of the State of Rio de Janeiro Dental School, Rio de Janeiro, Brazil and IASERJ Rio de Janeiro Brazil; ^2^ Private Practice Rio de Janeiro Brazil

**Keywords:** adhesives, autoclaving, implant‐supported restoration, resin cements, self‐adhesive resin cement, zirconia retention

## Abstract

**Purpose:**

The cement interface is particularly important for successful zirconia–titanium base (Ti‐base) restorations, as retention relies primarily on adhesive bonding. The aim of this in vitro study was to assess and compare the influence of a universal resin cement used with either a self‐adhesive or adhesive bonding protocol versus adhesive resin cements on the retention of zirconia to Ti‐base abutments, with and without autoclaving.

**Materials and Methods:**

Zirconia buildups were cemented to titanium‐base abutments using RelyX Universal (RXU) as a self‐adhesive resin cement, RXU with a primer (RXU/SUP), Panavia V5 (PV5) with primer, or multilink hybrid abutment (MHA) with primer. Half of the specimens were autoclaved. Push‐out testing was performed, and data were statistically evaluated using the analysis of variance (ANOVA), Tukey honest significant difference test, and family‐wise error rate method.

**Results:**

Of the nonautoclaved groups, RXU/SUP showed the highest initial mean push‐out load (1576.45 ± 195.86 N), followed by MHA (1268.10 ± 160.67 N), RXU (959.66 ± 139.24 N), and PV5 (905.84 ± 298.38 N). Autoclaving did not have a significant influence on cement push‐out load when compared directly within cement pairs. The push‐out load of RXU used as self‐adhesive cement was similar to PV5 with primer. Retention of RXU/SUP and MHA groups was significantly higher than that of RXU or PV5.

**Conclusion:**

In this in vitro study, RXU performed as well as PV5 groups and required the fewest preparation steps, suggesting it may be a good option for improving workflow efficiency. Results indicated a marginally positive effect of autoclaving between pairs, however, it was not significant.

A zirconia crown or framework cemented to a titanium base (Ti‐base) abutment is a standard reconstruction choice for implant‐supported restorations.^1^, ^2^, ^3^ Advantages of this hybrid concept include good marginal and internal fit, reproduction of a tailored emergence profile, high fracture strength, esthetic quality, retrievability, stability, and biocompatibility.^1^, ^2^, ^4^, ^5^, ^6^ A major benefit of extraoral cementation of the restoration onto the abutment is that it allows for complete removal of excess cement to eliminate the possibility of cement extrusion into the soft tissues.^7^

The cement interface is of particular importance for successful zirconia‐Ti‐base restorations, especially as retention relies primarily on adhesive bonding.^8^ A systematic review and meta‐analysis demonstrated a high short‐term survival rate of Ti‐base abutment restorations (98.6%), but technical complications were reported in numerous studies.^9^ The main technical problem leading to a remake of the restoration was debonding to the Ti‐base abutment.^9^ Bond strength has previously been shown to be influenced by the abutment/restorative material and cement type,^10^, ^11^, ^12^ as well as height and convergence angle,^13^, ^14^ prostheses adaptation, and surface pretreatment.^15^, ^16^

For bonding zirconia to a titanium abutment, self‐adhesive resin cements are an established simplified alternative to adhesive resin cements that require priming, but there is limited scientific evidence regarding the comparative bond strength of self‐adhesive versus adhesive resin cements to hybrid Ti‐base reconstructions.^4^, ^5^, ^17^ Evaluating the effect of autoclaving on bond strength for any resin cement indicated for extraoral cementation of zirconia buildups on titanium bases has also been advised.^18^ Although sterilization protocols have been suggested prior to prostheses installation,^8^, ^19^, ^20^ the use of resin cements has raised some concerns about their hydrothermal aging resistance since damaging effects of bond strength have been reported.^19^, ^21^ However, a systematic review and meta‐analysis on resin cement bond strength showed almost no effect of artificial aging when the bond surface was pretreated, such as sandblasted, and/or functional acidic monomers coated.^22^

A literature review revealed two relevant studies that evaluated the bond strength between zirconia and Ti‐bases. Pull‐out test data from one study evaluating the effect of resin cement protocol and autoclave sterilization on retention of zirconia crowns to Ti‐base abutments demonstrated higher retention for adhesive resin cement relative to self‐adhesive resin cement (*p *< 0.001).^16^ Although autoclave sterilization maximized bond strength for the self‐adhesive cement group, no significant difference was observed for adhesive resin cement groups. A previous push‐out study by Lang et al.^18^ assessed the bond strength between zirconia buildups and Ti‐bases among five different resin cements, all of which were used adhesively with their respective primer. Results showed significant differences in bond strength (7.6–33.7 MPa) among the five resin cements and a negative influence of autoclave sterilization on three of the cements.

The aim of this in vitro study was to assess and compare the influence of a universal resin cement used with either a self‐adhesive or adhesive bonding protocol versus adhesive resin cements on the retention of zirconia to Ti‐base abutments, with and without autoclaving. The tested null hypotheses were (1) resin cement material does not affect bond strength, and (2) autoclaving has no effect on bond strength for each material type.

## MATERIALS AND METHODS

Push‐out bond strength testing was performed to determine the retention of 40 zirconia buildups to Ti‐base abutments across four different cementation protocols. Half of the specimens were autoclaved. Bond strength data measured in newtons were statistically evaluated and compared.

**FIGURE 1:**
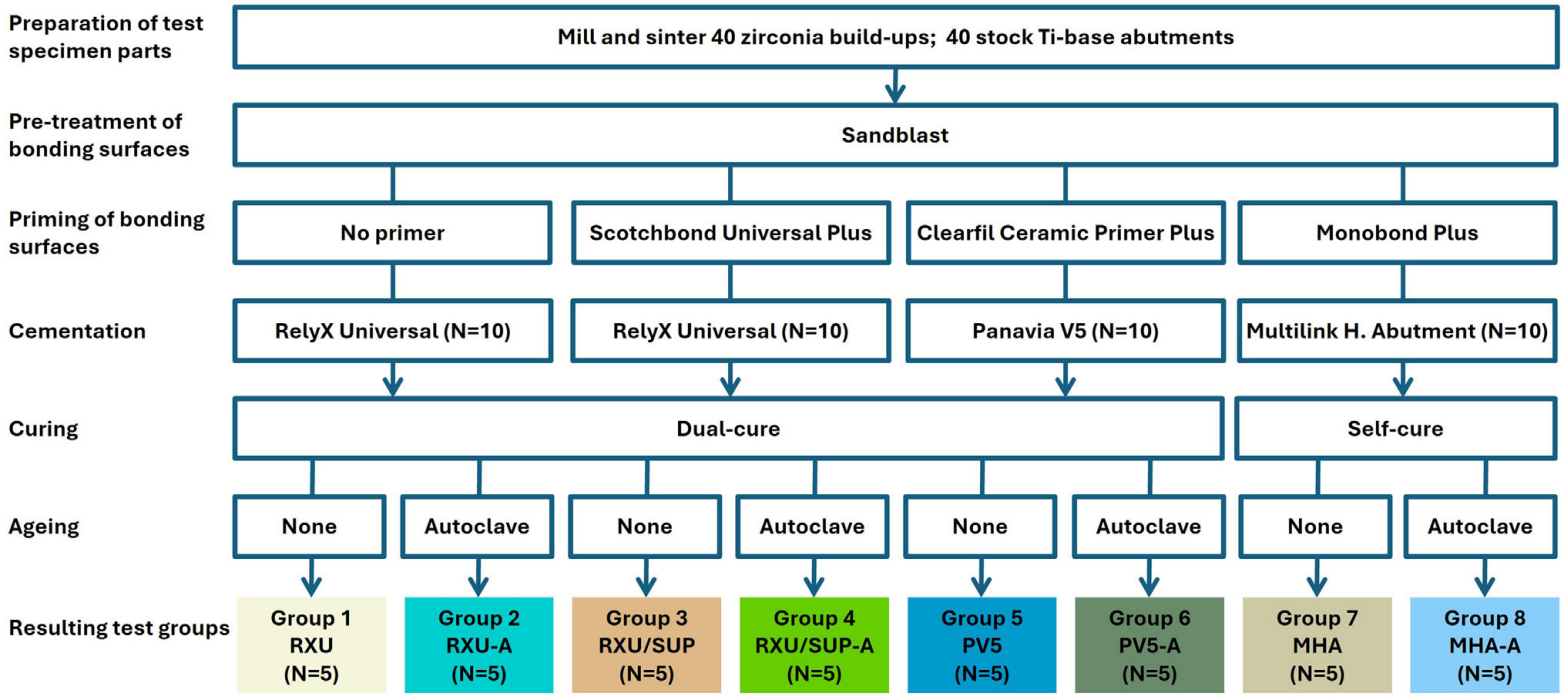
Steps for preparation of test specimens per test group.

Forty zirconia blocks were procured from one manufacturer (inCoris Zl meso L, Dentsply Sirona) and 40 Ti‐base L abutments were obtained from another manufacturer (EFF Dental Components). The zirconium‐oxide blocks contained prefabricated holes matching the abutment shape. Steps for preparation of test specimen parts are shown in Figure 1. To produce the zirconia buildups, blocks were milled (Primemill, Dentsply Sirona) to form a thinner (5.02 mm) specimen to facilitate testing and allow for pushing out the Ti‐base. The milled specimens were then sintered within a ceramic furnace (Speedfire, Dentsply Sirona), per the proposed workflow with Cerec. The sintering cycle was automatically assigned by the furnace system based on the milling job. By design of the CAD‐CAM system, there was no manual control over the cycle; it was system‐selected for ideal processing.

The specimens were randomly divided into four sets, with 10 sample buildups and 10 bases in each set. Cement and adhesive materials used for each group are listed in Table 1. RelyX Universal Resin Cement (RXU; 3M) was tested as a self‐adhesive resin cement and in combination with 3M Scotchbond Universal Plus Adhesive as a primer (RXU/SUP; 3M). Panavia V5 (PV5; Kuraray Noritake) and Multilink Hybrid Abutment (MHA; Ivoclar) were tested with their respective primers. Half of the specimens for each cementation protocol were assessed after autoclaving, and half were assessed without autoclaving, resulting in eight test groups. Sample size determination for each group was based on pilot testing that identified a narrow spread of values.

**TABLE 1:** Cement type and adhesive material per group evaluated.

Groups	Cement material	Cement Lot #	Adhesive/ Primer material	Adhesive/ Primer Lot #	Manufacturer
RXU 1,2	3M RelyX Universal Resin Cement	8974046	None	___	Solventum
RXU/SUP 3,4	3M RelyX Universal Resin Cement	8974046	3M Scotchbond Universal Plus Adhesive (no light cure)	7910510	Solventum
PV5 5,6	Panavia V5	4s0018	Clearfil Ceramic Primer Plus	1c0072	Kuraray Noritake
MHA 7,8	Multilink Hybrid Abutment	z05gkr	Monobond Plus	z05fr9	Ivoclar

### Sample preparation

The bonding surfaces of both the titanium abutments and the zirconia buildups were sandblasted (Microblaster Standard, Bio‐Art) with 50 µm aluminum oxide powder at 2.0 bar air pressure at a 1 cm distance for 20 s. The samples were cleaned in an ultrasonic bath with distilled water for 5 min. The bonding surfaces of both the titanium bases and the zirconia buildups were primed per manufacturer instructions for all specimens, except those in RXU groups (1 and 2), which did not require a primer.

A cementation protocol was performed for each of the tested dual‐cure resin cements according to the manufacturer's instructions for use, which recommended light‐cure followed by self‐cure. A uniform layer of the resin cement was applied to the pretreated surface of both the abutment base and the zirconia buildup. A customized pressure application device was used to seat each Ti‐base abutment to a zirconia buildup under a standardized force of 1.3 N. Excess cement was removed with a microbrush applicator (Microbrush Tube Series, Young Specialties) immediately after cementation.

Dual‐cured specimens (RXU, RXU/SUP, and PV5 groups) were cured by LED lights (Elipar DeepCure‐S, 3M) for 20 s per surface (total cure time: 4 sides × 20 s = 80 s) and self‐cured during dry storage for 24 h at room temperature. For MHA self‐cured specimens (Groups 7 and 8), a gel (Airblock, Dentsply Sirona) was applied around the specimens; after 10 min, the pressure was relieved and the specimens were washed off with distilled water, dried, and dry stored for self‐cure for 24 h at room temperature. Following the 24‐h storage, half of the specimens for each cement used (Groups 2, 4, 6, and 8) were autoclaved with a standard sterilization program for 30 min at a maximum temperature of 134°C. Dry cycle was 30 min followed by 10 min of cooling.

### Bond strength test set‐up

Push‐out testing was performed with a universal testing machine (Ultratester, Ultradent Products, Inc.) at a crosshead speed of 0.5 mm/min. The load required to push‐out each abutment was recorded in newtons.

### Statistics

Analysis of variance (ANOVA) models were conducted to compare the eight groups tested (RXU, RXU‐A (autoclaving), RXU/SUP, RXU/SUP‐A, PV5, PV5‐A, MHA, and MHA‐A). An interaction term was included and found to be nonsignificant. The F test was used to assess statistical significance. Tukey honest significant difference (HSD) post hoc test was performed to interpret the statistical significance of the difference among means by doing pairwise comparisons for all the groups. A compact letter display (CLD) statistical procedure was utilized to visualize the means and distributions compared from the ANOVA and Tukey HSD. Since these statistical tests involved multiple comparisons, a family‐wise error rate method was used to control for probability error. The adjusted significance level of α was $\alpha^{*}=1-\sqrt[{k}]{1-\alpha }$, where *k* is the number of pairwise comparisons. This method provided estimated mean differences and 95% confidence intervals for pairwise comparisons. R software (version 4.4.1, RStudio) was used for all statistical analysis; *p* < 0.05 was considered statistically significant.

## RESULTS

**FIGURE 2:**
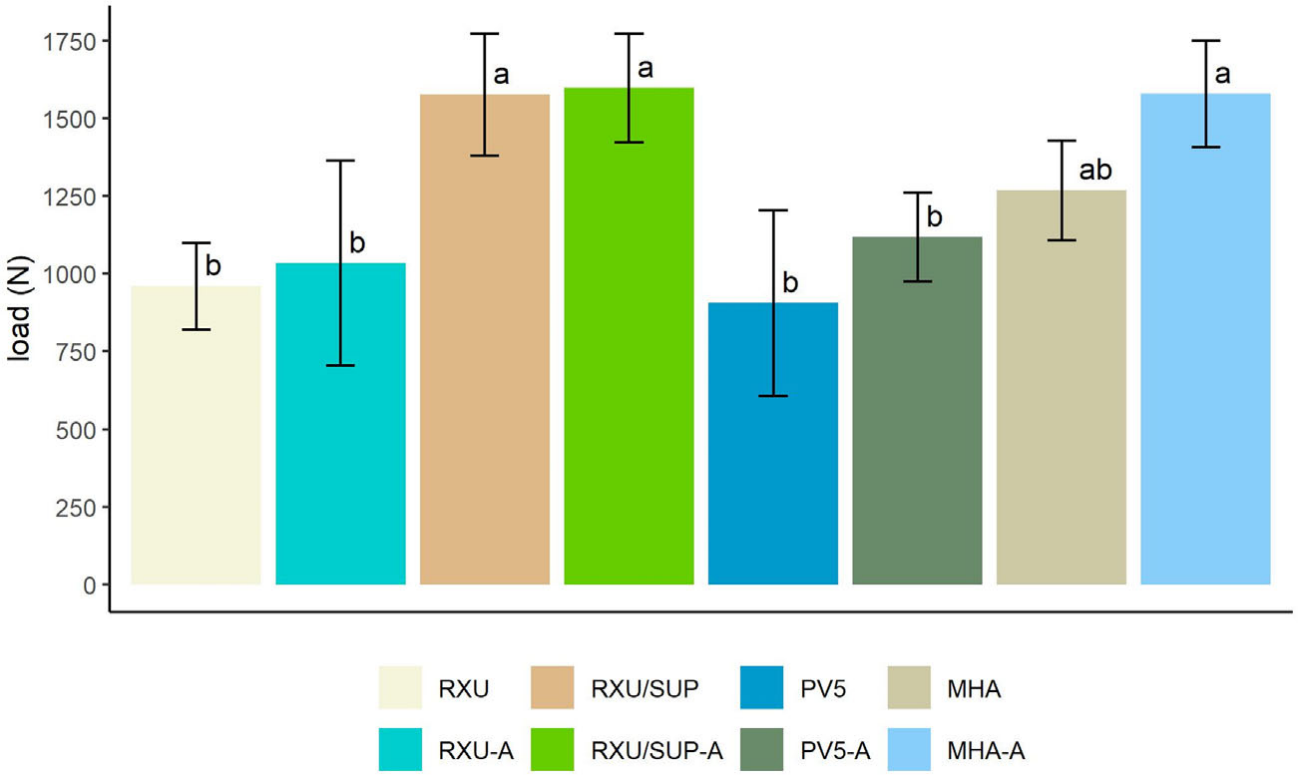
Push‐out test results and CLD statistical outcomes. Error bars represent estimated marginal mean ± one standard deviation error per group. Lower case letters (a, b) are the results of compact letter display (CLD) statistical procedure; means not sharing any letter are significantly different by the Tukey test at the 5% level of significance. N: newtons.

The one‐way ANOVA found statistical significance (*p* < 0.0001), indicating that at least one of the eight groups is different from the other groups. Figure 2 presents a comparative graph of mean push‐out load values, including statistical results from the CLD analysis.

Of the nonautoclaved groups, RXU/SUP showed the highest initial mean push‐out load (1576.5 ± 195.9 N), followed by MHA (1268.1 ± 160.7 N), RXU (959.7 ± 139.2 N), and PV5 (905.8 ± 298.8 N). In all pairwise tests, autoclaving did not have a significant influence on RXU, RXU/SUP, PV5, or MHA push‐out load when compared directly within cement pairs (*p*‐value = 0.427). However, with all data combined, autoclaving had a significant positive effect on bond strength overall (estimated mean difference: 155.0, 95% CI: [18.9, 291.2], *p*‐value = 0.028). The push‐out load of RXU used as a self‐adhesive cement was similar to PV5 with primer (PV5 vs. RXU estimated mean difference: −53.82, 95% CI: [−489.24, 381.59], *p*‐value = 0.999). The retention of RXU/SUP and MHA groups was significantly higher than that of RXU or PV5 groups. The largest differences were seen between RXU/SUP and RXU/SUP‐A versus PV5 (PV5 vs. RXU/SUP estimated mean difference: −670.61, 95% CI: [−1106.03, −235.20], *p*‐value < 0.001; PV5 versus RXU/SUP‐A estimated mean difference: −692.41, 95% CI: [−1127.83, −256.99], *p*‐value < 0.001).

## DISCUSSION

This present study is the first published in vitro analysis evaluating the zirconia‐Ti‐base bond strength of RXU used as a self‐adhesive resin cement and comparing it to the bond strength of RXU used as adhesive resin cement with SUP and two other established adhesive resin cements. The results displayed significantly higher bond strength values with RXU/SUP and MHA versus RXU (self‐adhesive) and PV5, both with and without autoclaving. Based on these findings, the null hypothesis that bond strength would not be affected by resin cement type was rejected (*p*‐value < 0.001). Absolute retention value of RXU/SUP was higher than MHA without autoclaving, but the difference was not significant (MHA vs. RXU/SUP estimated mean difference: −308.35, 95% CI: [−743.77, 127.07], *p*‐value = 0.328). When compared directly within cement pairs, autoclaving had a nonsignificant positive effect on RXU, RXU/SUP, PV5, and MHA push‐out load (*p*‐value = 0.427). Therefore, the null hypothesis that bond strength would not be influenced by autoclaving within the four cement pairs could not be rejected.

For this study, the cementation protocol was performed for each of the two tested dual‐cure resin cements according to the manufacturer's instructions for use, which recommended light curing for 10 s per surface. To partially account for the light attenuation by the thick zirconia specimens, the light‐cure time was doubled to 20 s, followed by a 24 h waiting period before testing to allow ample time for self‐curing. The high retention values obtained for the dual‐cure resin cements tested confirm that sufficient cure was achieved.

Compared to Bergamo et al.^16^ who showed significantly higher retentiveness of sandblasted self‐adhesive cemented Ti‐base abutments with autoclave sterilization, and Lang et al.^18^ who reported a negative effect of autoclave sterilization on three of the five cements tested, the present results indicated a marginally positive effect and no negative effect of autoclaving for any of the cement groups tested.

The decision to use a sample size of five specimens per group was informed by previous experience with bond strength push‐out testing, which incorporates factors that contribute to high repeatability such as two premachined parts made of standardized materials and a controlled strain application method by two well‐trained operators. Consistent with previous studies, the spread of values observed during pilot testing for this study was not wide, and therefore it was determined that testing more than five samples per group was unnecessary.

However, it should be noted that with this small sample size, this study may be underpowered for detecting smaller effects, particularly with respect to autoclaving. Specifically, the results showed that in all pairwise comparisons, autoclaving did not have a significant influence on RXU, RXU/SUP, PV5, or MHA push‐out load when compared directly within cement pairs (all *p*‐values were 0.32 or larger). Nonetheless, with all data combined, autoclaving demonstrated a significant positive effect on overall bond strength (estimated mean difference: 155.02, 95% CI: [18.86, 291.18], *p*‐value = 0.027). The nonsignificance within the individual groups is most likely attributed to the relatively small sample sizes; with the groups combined, the sample size was large enough to detect a significant effect of autoclaving.

The push‐out method was chosen to measure the retention force of zirconia cemented to titanium. The push‐out strength test was first advocated in 1970^23^ and has been widely used to evaluate the retention of resin cements in implant‐supported restorations.^18^, ^24^ The reported absolute push‐out load values were dependent on methods and measures used for this specific study, and thus results should be considered as relative comparisons and not directly comparable with absolute values in other studies. Accordingly, the bond strength of MHA has been similarly ranked higher than PV5 in a previously published push‐out bond strength study.^18^ Pull‐out data results from Bergamo et al.^16^ demonstrated higher retention values for adhesive resin cement associated with universal adhesive relative to self‐adhesive resin cement. In this present study, while push‐out bond strength of self‐adhesive RXU was lower than RXU/SUP and MHA, the bond strength of self‐adhesive RXU was similar to PV5 (with a primer).

During preparation of the specimens, the investigators observed smoother flow, reduced stickiness, and greater ease wiping off excess with RXU compared to the other cements, but none of these traits contributed directly to the study results.

Although this methodology allowed for the evaluation of individual variables, such as geometry, surface pretreatments, cement types, and adhesion protocols on the strength of a restorative system, it did not accurately reproduce all oral conditions that can cause dislodgement from multidirectional forces or other factors. In particular, specimens did not undergo cyclic fatigue testing or thermocycling (long‐term artificial aging), which could significantly affect degradation rate and bond strength across any of the groups.^21^ Hence, generalizing these in vitro results to clinical situations should be done with caution, and the data should be considered as initial bonding data, ranking the highest immediate bonding scores for different resin cement types with or without autoclaving.

## CONCLUSION

This study assessed the bond strength of a universal resin cement used as a self‐adhesive (RXU) versus an adhesive (RXU/SUP), compared to two other adhesive resin cements (MHA and PV5) in retaining zirconia to Ti‐base abutments, with and without autoclaving. The RXU/SUP‐A group displayed the highest mean bond strength, which was similar to the bond strength of the MHA‐A group. RXU performed as well as the PV5 groups yet did not require a priming step for preparation, suggesting it may be an efficient option for optimizing workflow without a tradeoff in performance. The results demonstrate the versatility of universal cement RXU, showing that it achieves bond strength comparable to the industry standard PV5 without the need for a primer, and matches the strength of specialized MHA when a primer is used. The results also indicated that autoclaving had no negative influence on bond strength for any of the cements under the specific conditions tested, which may be helpful in decision‐making where autoclaving is being considered. These preliminary findings on bond strength and autoclaving effects may provide valuable insights to guide future clinical studies on implant‐supported prostheses.

## CONFLICT OF INTEREST STATEMENT

Carlos Eduardo Sabrosa is a paid consultant of Solventum.
